# Advancing stroke genetics in Hawai‘i and the Pacific Islands

**DOI:** 10.3389/fstro.2023.1114785

**Published:** 2023-08-17

**Authors:** Stacy C. Brown, Christine Anne T. Galang, Mālialani Kana'iaupuni, Leah Dowsett, Keolu Fox, Kazuma Nakagawa

**Affiliations:** ^1^Neuroscience Institute, The Queen's Medical Center, Honolulu, HI, United States; ^2^John A. Burns School of Medicine, University of Hawai‘i, Honolulu, HI, United States; ^3^Indigenous Futures Institute, Institute for Genomic Medicine, Halıcıoǧlu Data Science Institute, Climate Action Lab, University of California San Diego, San Diego, CA, United States

**Keywords:** stroke genetics, Native Hawaiian and Pacific Islander health, population disparities, precision medicine, community based participatory research

## Abstract

Stroke, the second leading cause of death worldwide, has partially heritable risk. Genome-wide association studies (GWAS) of stroke continue to identify increasing genetic risk loci. These discoveries point to novel disease mechanisms and causal risk factors, and herald genetics-based precision medicine strategies. In Hawai‘i, people of Indigenous communities who identify as Native Hawaiian or Pacific Islanders present with stroke at younger ages and suffer dramatically higher stroke mortality rates compared with other regional populations. This disparity is compounded by relative ancestral underrepresentation in stroke genetics research and, by extension, exclusion from cutting-edge medical opportunities based on genetic discovery. In this article, we discuss the issues contributing to the scientific biases experienced by Indigenous populations in the Pacific Islands, as well as community-based efforts now underway to address them.

## Health disparities amidst diversity

Across the world, stroke is the second-leading cause of death and the third-leading cause of death and disability combined (GBD 2019 Stroke Collaborators, 2021). Although stroke is globally prevalent, epidemiological studies have also established that the burden of stroke is unequally distributed across racial and ethnic groups (Elkind et al., 2020). Studies focusing on disparities experienced by Black and Hispanic populations comprise the largest body of evidence (Nakagawa et al., 2013), but the public health problems posed by health disparities affect many more groups of people who have experienced social, economic, and environmental disadvantage based on racial, ethnic, and/or Indigenous identity.

Hawai‘i leads the country in terms of racial and ethnic diversity (US Bureau, 2010). U.S. census data has the racial and ethnic makeup of Hawai‘i residents at just over one third Asian, one quarter identifying with two or more races, and another one quarter white. Hispanic and Black people comprise 11.1% and 2.2%, respectively (Narcisse et al., 2021; US Bureau, 2022). Census statistics have Native Hawaiian or Pacific Islander (NHPI) people comprising 10.5% of the state's population.

However, diversity alone does not level the field for health equity. Locally, NHPI's bear a disproportionate burden of cardiovascular disease, vascular risk factors, and mortality related to cardiovascular disease (Fukino et al., 2007). Mortality rates are four times as high, and NHPI are 1.7 times more likely to die from heart disease in particular, when compared to white individuals (Pan et al., 2019). They are more than twice as likely to develop obesity and type 2 diabetes (Biffi et al., 2011; Kim et al., 2015; Marini et al., 2018). Our previous research found that among stroke patients admitted to Hawai‘i's major tertiary stroke referral center, NHPI patients who presented with ischemic stroke were nearly a decade younger and were more likely to have diabetes, hypertension, and obesity (Nakagawa et al., 2013). NHPI ischemic stroke patients were also less likely to receive treatment with intravenous tissue plasminogen than white patients (Nakagawa et al., 2017). Among hemorrhagic stroke patients, NHPI individuals presented more than a decade younger than their white counterparts (Nakagawa et al., 2012).

The first step to addressing population health inequities is reconciling a range of deficiencies in how race/ethnicity data are collected, reported, and used. Early attempts at establishing population specific trends in precision medicine are confounded by non-standardized and aggregated data collection methods (Kamaka et al., 2021). In 1997, the Office of Budget and Management Standards for the Classification of Federal Data on Race and Ethnicity separated the “Native Hawaiian or Other Pacific Islander” category from the “Asian” category for the first time (US Bureau, 2000; Office of Management and Budget, 2021). Around 90% of people identifying as Kānaka Maoli (Native Hawaiian, here referring to the original inhabitants of the Hawaiian archipelago) are of mixed race, making the actual percentage of self-identifying Kānaka Maoli closer to 22% (Hawaii Health Data Warehouse, 2011; Nguyen and Florentina, 2015; Kana'iaupuni et al., 2021; State of Hawaii Department of Business, Economic Development and Tourism, 2021). Another 4% of the state's population is comprised of a diverse community of Pacific Islanders, here referring to the original inhabitants of the islands of Oceania. Today, NHPI communities combined are one of the fastest growing and most diverse racial groups in the U.S. (Advancing Justice LA, 2014).

While social determinants of health, such as access to health care, housing quality, employment, education, environmental exposures, and structural racism (Cogburn, 2019), must be addressed to create more equitable health systems in Hawai‘i; disease risks and responses to treatments that relate to genetic and epigenetic variation may be addressed by research that uses data on genetic ancestry, genotypes, and biomarkers. Race, ethnicity, and Indigeneity capture different information than genetic ancestry, with the former shaped by geographic, cultural, and sociopolitical forces and the latter reflecting the geographic origins of a population. That said, these constructs inextricably correlate, such that informed use of genetic analyses may be harnessed toward efforts to reduce health disparities among racially identified groups now and into the future (Borrell et al., 2021).

## Implications of Kānaka Maoli underrepresentation in genetic and genomic research

Currently 95% of large-scale screens of human genetic variation and 95% of clinical trials—and thus the future of precision biomedicine (i.e., personalized, predictive, and preventive medicine)—feature only individuals of European ancestry (Mills and Rahal, 2020). This has resulted in biased genetic algorithms and failure to identify disease-associated variants with health implications for underrepresented communities, including Indigenous Peoples in the Pacific. In other words, there is and will continue to be a lack of equity in the utility of precision medicine strategies.

For many monogenic conditions, variants associated with disease in non-European ancestry are not well-characterized and thus genetic testing is of limited utility. Because populations of Hawaiian ancestry are nearly excluded from large databases used to evaluate allele frequency relative to disease prevalence, novel variants identified in populations of non-European ancestry are likely to be classified as variants of uncertain significance.

Kānaka Maoli are also at risk to receive treatment recommendations that do not consider important loss of function variants that impact drug metabolism, and which are less commonly found in populations of European ancestry. An example was illustrated in a lawsuit filed by the state of Hawai‘i against Bristol-Myers-Squibb and Sanofi-Aventis, which manufacture the antiplatelet drug clopidogrel (Abercrombie and Louie, 2014). In the field of stroke, randomized controlled trials established the efficacy of dual antiplatelet therapy (DAPT) with aspirin and clopidogrel to prevent recurrent ischemic stroke in patients with non-cardioembolic minor stroke or high-risk transient ischemic attack (Wang et al., 2013). However, subgroup analysis of one of these trials found no benefit of DAPT in carriers of a CYP2C19 loss of function allele (Wang et al., 2016). CYP2C19 is the primary cytochrome P450 enzyme involved in metabolism of clopidogrel to its active metabolite and has arisen as the factor most highly associated with interindividual variability in response to clopidogrel therapy (Brown and Pereira, 2018). An estimated 30% of Kānaka Maoli have loss of function mutations in CYP2C19. Loss of function variants of CYP2C19 have also been found to be markedly more prevalent in Asian populations (Shuldiner et al., 2009). Despite these variants rendering the drug ineffective in the prevention of strokes and myocardial infarctions, the medication was marketed for widespread use without genotype testing guidance.

## Prospects of inclusive research

Over the last decade, stroke genetic and genomic research has benefited from rapid advances in genotyping technology and large international collaborations, leading to exponential progress in the identification of polymorphisms that influence cerebrovascular risk. Before the first large GWAS was published in 2007 (The Wellcome Trust Case Control Consortium, 2007), population genetics focused on candidate-gene studies of rare mutations that cause mendelian disorders. Since then, an ever-increasing number of single nucleotide polymorphisms (SNPs) associated with stroke risk have been identified through hypothesis-free interrogation of the human genome (Biffi et al., 2010; Woo et al., 2014; Malik et al., 2018; Mishra et al., 2022). To date, 89 stroke risk loci have been identified (Falcone and Rosand, 2016; Malik et al., 2018; Mishra et al., 2022). Risk loci have been identified for all major stroke subtypes, and although several of these integrate into previously understood biological mechanisms for stroke, roughly a third point to novel pathophysiological pathways (Dichgans et al., 2019).

There is increasing recognition that multi-ancestral participation in genetic and genomic research is crucial to discovery that has implications for all populations. One example in vascular disease is the identification of rare loss of function mutations in the proprotein convertase subtilisin/kexin type 9 (PCSK9) serine protease gene associated with significant reductions in low-density lipoprotein (LDL) cholesterol levels. This was discovered in a study of genetic variants in Black participants in the Dallas Heart Study, and it led to the development of highly effective LDL-lowering drugs (Cohen et al., 2005). Because the variants are of higher frequency in people of African ancestry, the inclusion of Black participants in the study was crucial to their identification. More population-specific variants with potential medical actionability are anticipated to be identified as studies include more understudied populations.

Growing numbers of known disease susceptibility loci further allow the creation of polygenic risk scores, which can heighten our ability to stratify risk and determine which individuals will benefit most from targeted interventions. Polygenic risk scores provide a powerful tool that aggregates information about multiple SNPs, each with small effects, to examine the cumulative predictive ability of genetic variation at known loci on disease outcomes and related phenotypes (Smith et al., 2015). Polygenic risk scores can be calculated as the sum of risk alleles for a given person and provide a disease risk prediction tool with demonstrable advantages over approaches using only established clinical risk factors (Khera et al., 2018; Acosta et al., 2020). However, previous studies showed that polygenic risk scores for common diseases had better accuracy when target and discovery cohorts are ancestry-matched (Majara et al., 2021).

Genetics can also accelerate drug discovery. Recent studies indicate that selecting genetically supported treatment targets doubles the success rate of clinical intervention development and obtaining regulatory approval (Hurle et al., 2016). Genetics can help anticipate the full range of safety and efficacy consequences of pharmacological interventions and prioritize targets for clinical trial exploration. Again, multi-ancestry studies will be essential for the development of genetics-derived treatment strategies that are effective across populations.

## Special considerations for research involving Kānaka Maoli

Important obstacles to research inclusive of Indigenous populations persist such that even actively enrolling, large-scale genome sequencing initiatives are limited by the absence of Kanaka Maoli participants (Fox, 2020). Important concerns are lack of community participation in research agenda-setting, lack of benefit to the communities contributing data, and failure to appropriately obtain individual and collective consent. These issues impact the willingness of Kānaka Maoli to participate in biomedical research. In 2003, the Paoakalani Declaration was created from a gathering of Kanaka Maoli elders, artists, cultural and spiritual leaders, academics and attorneys. It reinforced Kanaka Maoli worldviews and concepts of ancestral genealogy, rights over traditional knowledge, and the sacred and inalienable nature of Kanaka Maoli biologic and genetic materials. The Kanaka Maoli community urged the cessation of university intentions to map the Hawaiian genome and condemned medical research on their biological specimens as acts of biopiracy and biocolonialism (Association of Hawaiian Civic Clubs, 2004; Office of Hawaiian Affairs, 2018).

Foundational work on NHPI interests in biological research has established several important concepts: (1) the historical failure of traditional research institutions to protect Indigenous peoples from group harm (Chang and Lowenthal, 2001; Kaiser, 2019); (2) the preference for tailored consent and disclosure processes among Kānaka Maoli participating in research (Fong et al., 2006; Braun et al., 2014; Tauali‘i et al., 2014); and (3) robust community interest in participating in health studies, so long as the research is undertaken with explicit concern for and involvement of the participating communities (Vawer et al., 2013). Prior community based participatory research which engaged Kānaka Maoli in discussions around biobanking identified genetic and biological research as one of the five priority issues for awareness and research (Tauali‘i et al., 2014).

**Figure 1:**
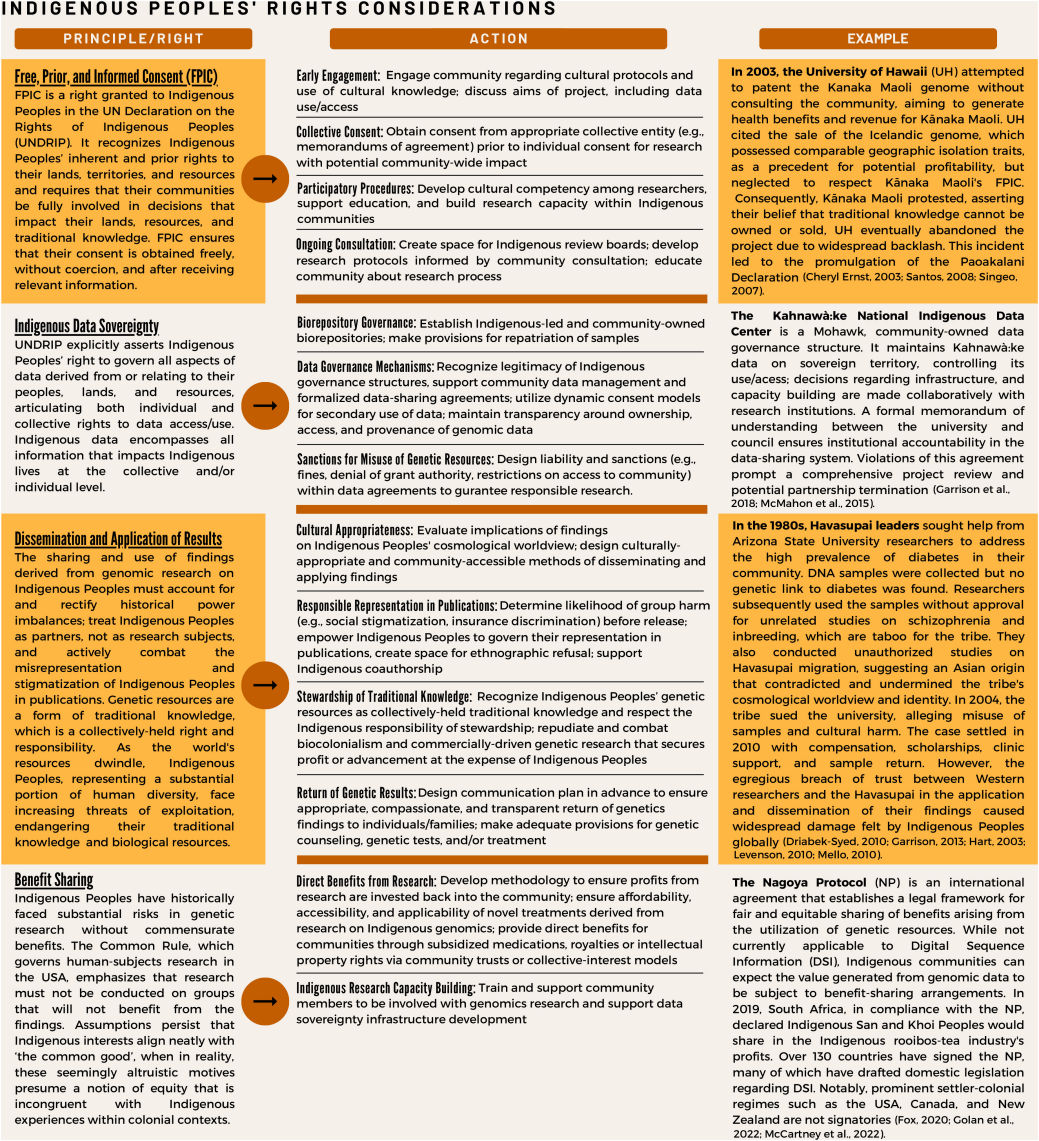
Key research principles concerning Indigenous peoples' rights, with example cases and strategies for addressing.

Successful efforts to increase Kanaka Maoli representation in genetic and genomic research require that they are collaborators in the development of research questions and participants in the governance of biological and derived data. Community interests, needs and cultural values must be incorporated into research priorities. Education, training, and sustainable development must be cornerstones of the consent and research protocols. Many Indigenous communities favor early engagement, participatory procedures, and ongoing consultation throughout the research process with expectations of reconsent for future research (Ward, 2011). Additionally, especially for genetic and genomic research, in which findings can affect entire populations, an established best practice is to seek prior agreement from the community through its representatives before seeking informed consent from its individual members (Garrison et al., 2019). A challenge that is particular to establishing collective consent among Kānaka Maoli is the community's lack of a recognized, centralized governance structure. Widespread and recurrent engagement community engagement is thus vital to research progress. Principles for consideration of Indigenous rights in biomedical research are further summarized in Figure 1.

## The way forward

Genetic and genomic research endeavors must respect Indigenous Peoples' knowledge and culture, protect data sovereignty, support capacity building, and determine transparent research priorities in partnership with communities to ensure that research benefits the populations involved (Santos, 2008). Building a system to achieve these goals is multi-faceted and requires a multidisciplinary team led by Indigenous scientists and healthcare providers. The first successful example of such an initiative in the United States is the Native BioData Consortium (NBDC), a 501(c) (3) non-profit research institute located on Sioux territory in the Great Plains and led by Indigenous scientists and tribal members. Its goal is to use health data for quality-of-life improvement and to ensure that advances in genetics and health research benefit all Indigenous people. An analogous effort based in Hawai‘i is imperative to ensuring inclusive future research that will bring parity and equity to the Lāhui (the Hawaiian Kingdom).

Ensuring representation amongst the NHPI communities can prove to be challenging, as the Pacific Islander cohort can include over 30 distinct groups across Oceania. Given the breadth of this diverse group of peoples, we must be more mindful about accurately representing each of these communities as we move forward with precision medicine. The scientific community recognizes that accurate representation of these groups in genomic databases is the first step toward achieving parity. With proper informed consent and taking this ideology a step further, we propose that using DNA haplogroup information to inform and curate this database, rather than relying soley on self-reported or investigator-assigned ancestry, will help us understand how to best characterize each participant. In doing so, disaggregated genomic data will be a powerful tool that will allow for the discovery of impactful, targeted therapy that is clinically-relevant, culturally-sensitive, and inclusive to the diverse peoples of the Pacific.

**Figure 2:**
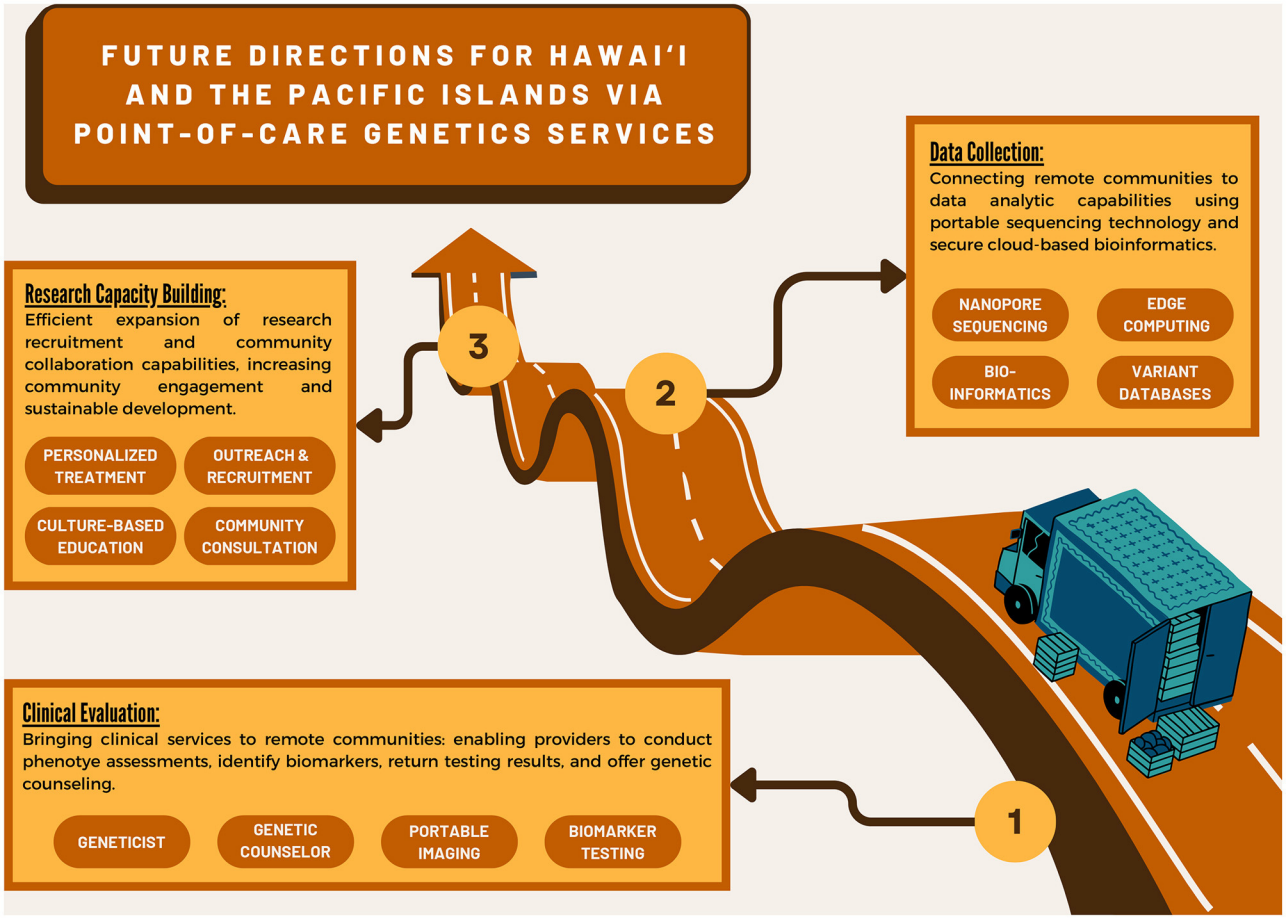
Infographic of future genetic research directions leveraging point-of-care technology and services.

To deliver precision medicine to some of the most isolated communities on the planet from Moananuiākea (the far-flung island archipelagos of the Pacific Ocean) to Turtle Island (First Nations communities across the Americas), we look to the potential of point-of-care access to healthcare. By making access to precision medicine more modular, portable, and customizable, we not only have the opportunity to de-black-box emerging technologies like next-generation sequencing; we also create space and allow for specialization around individual community health needs, thereby enabling us to address the complexity of health issues in historically marginalized communities. This approach also encourages user-led innovation and iteration, leading to continuous improvement in diagnostic outcomes for these communities (Figure 2).

Data is the most valuable resource on our planet, surpassing oil in 2018. Biomedical health data generated in a space where 95% of both large-scale screens of human genetic variation and clinical trials only include individuals of European ancestry is a huge value add opportunity. Generating new genome sequence data for “never been sequenced populations” will lead to the discovery of novel genetic mutations, and the identification of new bio-mechanisms that contribute to stroke and cerebrovascular disease susceptibility. This new intellectual property (IP), in the form of diagnostic data, could yield novel identification of bio-mechanisms and biomarkers that can inform the development of new forms of RNA-based gene therapy in the future (Fox, 2020). This “people-driven” therapeutics research and development strategy to identify new IP is especially helpful in new or never-been-sequenced founder populations that have unique migratory histories, like communities subsisting at high elevation in the Himalayas or those found in remote Oceania. Guided properly with active recognition of Indigenous data sovereignty, new technology can be used to address health disparities while also producing valuable IP for both the participants and the genomics research community. We have an opportunity here to provide precision medicine for those who need it; allow them to have ownership of their data; create equity in the development of new medicines; and expedite innovation in human biomedical research.

## Data availability statement

The original contributions presented in this perspective are included in the article/supplementary material, further inquiries can be directed to the corresponding author.

## Author contributions

All authors contributed to the generation of the manuscript idea, manuscript preparation and revision of the final manuscript. All authors contributed to the article and approved the submitted version.
